# Concise Review: Advances in Generating Hepatocytes from Pluripotent Stem Cells for Translational Medicine

**DOI:** 10.1002/stem.2368

**Published:** 2016-04-22

**Authors:** Dagmara Szkolnicka, David C. Hay

**Affiliations:** ^1^ Medical Research Council Centre for Regenerative Medicine, University of EdinburghEdinburghScotlandUnited Kingdom

**Keywords:** Pluripotent stem cells, Hepatocyte‐like cells, Cell‐based modeling, Technology scale‐up, Translational medicine

## Abstract

The liver is one of the major organs in the human body. Severe or prolonged exposure of the liver to different factors may cause life‐threatening disease, which necessitates donor organ transplantation. While orthotopic liver transplantation can be used to effectively treat liver failure, it is an invasive procedure, which is severely limited by organ donation. Therefore, alternative sources of liver support have been proposed and studied. This includes the use of pluripotent stem cell‐derived hepatocytes as a renewable source of cells for therapy. In addition to cell‐based therapies, in vitro engineered liver tissue provides powerful models for human drug discovery and disease modeling. This review focuses on the generation of hepatocyte‐like cells from pluripotent stem cells and their application in translational medicine. Stem Cells *2016;34:1421–1426*

**Significance Statement**

There has been tremendous progress in the development of efficient and defined hepatocyte differentiation from pluripotent stem cells, yet instability of hepatocyte cell phenotype still exists. We have shown that this is not specific to stem cell‐derived hepatocytes, but also observed in gold standard primary hepatocytes cultured in two or three dimensions 48. Therefore, we need to stabilize cell phenotype, so that somatic cell technology can be dependably scaled for application. Key to this will be the building of supportive liver niches in vitro. This review focuses on the new advances in the generation of hepatocyte‐like cells and their application in translational medicine.

## The Liver

The liver is a multifunctional and highly regenerative organ, playing an important role in human physiology 1. While resilient, the liver is susceptible to tissue damage and, therefore, degenerative diseases. Significant morbidity, mortality, and economic burden are associated with human liver disease. Therefore, the development of new systems that improve the study and treatment of liver disease are essential.

The structure of the liver is essential to its multifunctional performance. In the context of disease, liver structure becomes gradually more distorted with the loss of the hepatocyte compartment and consequently organ function 2. Hepatocytes are located in the parenchyma and comprise approximately 70‐80% of the liver mass 3. Their function is supported by the nonparenchymal cells, forming a functional unit termed the acinus 4. Hepatocyte polarization is essential for proper function. The basolateral surface of hepatocytes is directly connected with sinusoidal endothelial cells, which facilitate mass transport between the parenchyma and the blood stream. At the apical surface, tight junction formation between hepatocytes is required for canaliculus formation and bile acid transport 5.

Although hepatocytes are extremely stable in vivo, they rapidly lose their phenotype in vitro 6. This has significant consequences for scientists and clinicians who wish to build models of human liver biology “in a dish” or develop pioneering treatments for human liver disease.

## Cell‐Based Models

**Figure 1:**
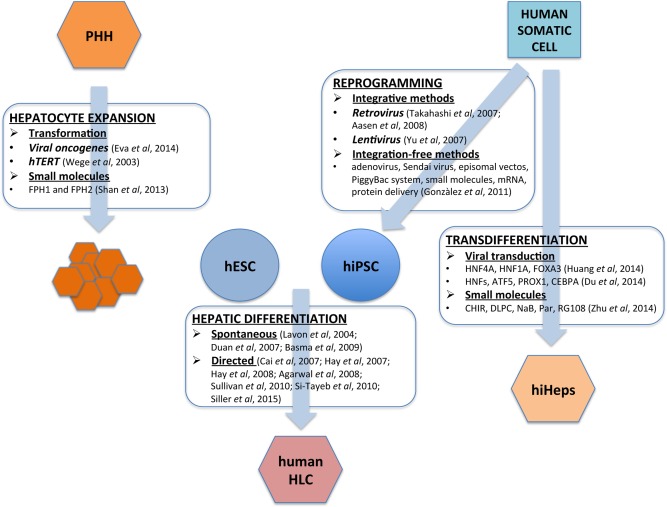
Hepatocyte differentiation and expansion. Hepatocytes can be derived from human pluripotent stem cells (HLC) and via conversion of human somatic cells (hiHeps). Hepatocytes can also be expanded from primary human hepatocytes (PHH) using cell transformation techniques and small molecules. Abbreviations: hESC, human embryonic stem cell; hiPSC, human induced pluripotent stem cell; PHH: primary human hepatocytes.

Although human hepatocytes are scarce and inherently unstable in vitro, they have been successfully deployed to model human biology and bridge patients until their liver recovers or a transplant becomes available 7. To bypass the issues of scarcity and instability, several groups have immortalized human hepatocytes (Fig. 1). Unfortunately, the derivative cell lines exhibited both poor function and karyotypic instability, limiting their large‐scale application (for a review see ref. 28).

Given the practical issues associated with primary material, researchers have turned to cancer‐derived cell lines to perform in vitro studies. Hepatic cancer cell lines such as HepG2, Hep3B, HepaRG, or Fa2N‐4 have been extensively used in drug metabolism studies. Although these cell lines have their uses, poor cell phenotype and their resilience to toxicological insult, limit their accuracy and extrapolation to human biology. To overcome the issues associated with cancer or immortalized cell lines, researchers have focused on improving hepatocyte cell expansion and phenotype using synthetic polymers 29 and small molecules 10, 24. More recently, Levy et al. 30 have expanded primary human hepatocytes, for up to 40 population doublings, by ectopic expression of Human Papilloma Virus (HPV) E6 and E7 oncoproteins. Although the cells produced in vitro will be useful for in vitro modeling studies, their utility is limited, as they are not derived from self‐renewing populations and are not appropriate for use in the clinic.

Human pluripotent stem cells (PSCs), human embryonic (hESCs), and induced pluripotent (hiPSCs) stem cells, offer a scalable alternative to primary and transformed cells 31. hESCs are derived from the inner cell mass of blastocysts that are unsuitable for human implantation. The cells display two important attributes, self‐renewal and pluripotency, promising an unlimited supply of human somatic cells in vitro 32, 33.

Induced pluripotent stem cells (iPSCs) were initially generated by the introduction of four transcription factors (Oct 3/4, Sox2, c‐Myc, Klf4) using integrative retrovirus technology 11, 34. This led to multiple genomic insertions and was a major concern for the field. Since those seminal studies there have been numerous attempts to generate insertion‐free human iPSCs, using Sendai virus 35, adenovirus 36, episomal vectors 37, the piggyBac system 38, 39 or mRNAs 40. Those methods have proved successful, with Sendai virus reprogramming system currently considered as the most efficient reprogramming system 41, 42.

PSCs have created new opportunities to model human biology and provide the prospect of personalized medicine. In recent years, significant progress has been made in differentiating PSCs into HLCs (Fig. 1). The use of defined factors and serum‐free media has facilitated the development of efficient procedures for HLC specification using either spontaneous or directed differentiation. Spontaneous differentiation involves formation of three‐dimensional (3D) multicellular aggregates, termed embryoid bodies. In the presence of particular growth factors (e.g., Wnt, BMP, FGF, HGF, or OSM) these 3D structures differentiate into HLCs 15, 16, 17. Although this method is reproducible, its spontaneous nature has several drawbacks, including low efficiency and mixed cell type generation, which limit technology scale‐up. In contrast, directed differentiation is performed in two dimensions and has proved to be more efficient 18, 19, 20, 21, 22, 23, 43, 44, 45, 46, 47, 48. Some of the achievements to date using HLCs include; the accurate prediction of human drug metabolism 49, 50, 51, the mechanistic analysis of drug‐induced liver injury 52, 53, the use of noncoding RNAs to modulate drug overdose 54, the study of virus infection and replication 55, 56, 57, 58, 59 and the ability to model inherited monogenic metabolic disorders of the liver 60, 61.

Such findings demonstrate the importance of PSC based liver models in the development of platform technologies to study human biology. This will likely lead to the identification of new medicines or the re‐purposing of existing medicines to treat human disease.

In addition to PSCs, somatic cells have been transdifferentiated to hepatocytes (iHeps). Like iPSCs, iHeps can be derived from defined genetic background. Importantly, the production of iHeps bypasses the need for pluripotency (Fig. 1). Initially, Huang et al. 62 and Sekiya & Suzuki 63 generated induced hepatocytes (iHeps) from mouse fibroblasts using viral transduction and expression of either GATA4, HNF1A, and FOXA3 and inactivation of p19^Arf^ or HNF4A in combination with FOXA1, FOXA2, or FOXA3. Three years later Huang et al. 25 and Du et al. 26 generated functional hiHeps from human somatic cells using viral transduction and expression of either FOXA3, HNF1A, and HNF4A or overexpression of hepatic nuclear factors (HNF1A, HNF4A, HNF6) in combination with ATF5, PROX1, and CEBPA. The same year, Zhu et al. 27 used a panel of small molecules to initiate hepatocyte differentiation from incompletely reprogrammed human fibroblasts. While the small molecules studies were of interest, the incompletely reprogrammed nature of the cells plus the cocktail of small molecules will complicate technology transfer and scale‐up. The issue of hiHep cell scale‐up has recently been addressed in an elegant study by Shi et al. 64. Excitingly, the scaled hiHep populations were deployed in an artificial liver device and corrected abnormal blood biochemistry following acute liver failure in pigs, offering a significant therapeutic potential for the future.

## In Vivo Transplantation of Liver Progenitors and Hepatocyte Like‐Cells

Currently, the only cure for advanced liver disease is donor organ transplant. Although highly successful, the lack of donors has forced scientists to look for the alternative sources of liver support. Hepatocyte transplantation has been used to successfully treat compromised liver function. However, routine access to good quality donor livers, as for organ transplant, remains a significant limitation. Therefore, the development of a scalable and renewable source of hepatocytes would be a game changing addition.

Although several studies have demonstrated successful transplantation of pluripotent‐derived hepatic cells in rodents 65, 66, the limited capacity of cell proliferation in vivo, poor engraftment, and immune rejection rates are major challenges to clinical application. To address this, Song et al. 67 have efficiently transplanted hiPSC‐derived cells in immunocompetent mice by pre‐engineering 3D cell coaggregates with stromal cells followed by hydrogel encapsulation. Nagamoto et al. 68 took a different approach, improving hepatocyte engraftment and animal survival by attaching the PSC‐derived hepatocyte sheets onto the surface of the liver during acute liver failure. In addition, noncoding RNAs have also been used to improve cell‐based therapies. In a study by Möbus et al. 69 miR‐199a‐5p inhibition in hESC‐derived HLCs enhanced cell engraftment in the liver.

Transplantation of adult hepatic progenitors is another promising cell‐based therapy. Recently, Lu et al. 70 transplanted hepatic progenitor cells (HPCs) from wild‐type mice to adult mice livers where the hepatocyte compartment had been conditionally deleted. Wild‐type HPCs successfully engrafted and expanded in vivo, restoring both hepatic and biliary compartments. In addition, Huch et al. 71 have demonstrated that murine Lgr5+ liver stem cells can be expanded as epithelial organoids using a Wnt agonist and subsequently differentiated into functional hepatocytes and bile ducts in vivo. Two years later, the same group reported the successful isolation, expansion, and differentiation of human bile duct‐derived progenitor cells 72.

## Culture Definition and Technology Scale‐Up

Human PSC biology has revolutionary potential for modern medicine. The majority of procedures, published to date rely on undefined and/or xenobiotic containing culture systems. The undefined components found in bovine serum, Matrigel^TM^, or from feeder cell layers, among others, elicit unknown biological effects and lead to phenotypic variability in vitro. This is a significant limitation, which hampers the scale‐up and application of PSCs and their derivatives 73.

To overcome these issues, researchers and companies have focused on developing defined, xeno‐free, and serum‐free media formulations 74, 75, 76, 77, 78, 79, 80, 81. In a multicenter trial, Akopian et al. 73 examined eight different serum‐free media formulations in five different laboratories and concluded that StemPro and mTeSR1 were the only formulations, which supported stem cell self‐renewal for at least 10 passages.

In addition to stem cell self‐renewal and the maintenance of pluripotency, defined culture is essential for cellular differentiation, scale‐up, and biomedical application. To improve the definition of the differentiation procedures, serum‐free processes have been developed 45 and used in combination with recombinant extracellular matrices 47. To improve culture definition and further reduce differentiation costs, small molecules have also been used to hepatic differentiation 19, 20, 24, 27. While promising, those small molecule studies relied on undefined culture components, demonstrating the need for further research and development in this space.

## Improving the Current State of the Art and Technology Scale‐Up

Many studies have focused on improving hepatocyte physiology and biology “in the dish.” Studies by Miki et al. 82 demonstrated that oxygenation as well as continuous supply of nutrients, using hollow fiber technology, promoted mature hepatocyte gene expression. Additional studies using defined media and synthetic tissue culture substrata, have shown promise in cell specification and the maintenance of hepatic phenotype from both research and GMP hESC lines 46, 47. These provide GMP ready options for large‐scale manufacture. In addition to culture definition, faithful markers of cell specification are required to ensure proper cellular differentiation and to control for quality. Currently, the most widely used markers for tracking endoderm induction from PSCs are C‐KIT, CXCR4, and EPCAM 14, 83. Although efficient, these markers are not endoderm specific. Therefore, new lineage markers are required to track endoderm and ultimately hepatic specification.

Recently, Holtzinger et al. 84 have identified two new endodermal markers, HDE1 and HDE2. HDE1 marked the component of the definitive endoderm population with high hepatic potential, whereas HDE2 tracked developing hepatic progenitors and hepatocytes.

Those markers are important additions to the field and allow for the formation of purer DE population from stem cells, improving cell identity and phenotype in culture. Along similar lines, Kido et al. 85 have shown that carboxypeptidase M is an efficient marker to isolate and culture hepatic progenitors from PSCs. In addition to cell tracking, the identification of gene signatures, which predict stable cell phenotype are also important. Recently, we demonstrated that the use of a defined polymer substrate, in conjunction with serum‐free hepatic differentiation, revealed a unique gene signature (MMP13, CTNND2, and THBS2), which predicted stable hepatocyte performance from both research and GMP hESC lines 46. In the future, gene signatures may serve as important criteria for large‐scale manufacture and product release.

In addition to directing and monitoring cell differentiation, it is important to provide a supportive niche, which transmits key stimuli to support somatic cell phenotype. While two dimensional hepatic differentiation systems are efficient and functional, they are not equivalent to freshly isolated human hepatocytes 47, 48. To overcome these issues, and mimic 3D tissue architecture, efforts have focused on developing new in vitro platforms using natural and/or synthetic materials, fluid flow and bioprinting 29, 86, 87, 88, 89. Excitingly, hiPSC‐based systems have also been shown to generate functional and implantable human liver tissue 90.

In the quest for large‐scale automated tissue production, bioprinting is an attractive approach. Recently, Faulkner‐Jones et al. 88 bioprinted hiPSC‐derived hepatocytes in a 3D alginate matrix. The printed cells survived this process and expressed the hepatic markers hepatocyte nuclear factor 4 alpha, albumin, and zona occludin 1. While these studies are encouraging, future experimentation should extensively characterise the performance of bioprinted tissue in vitro and in vivo.

Bioinformatics has also proved highly effective in studying cell specification and phenotype. We have recently performed a genome‐wide study where PSC‐derived hepatocytes were compared to freshly isolated and cultured primary human hepatocytes (PHHs) 48. In these studies, we identified unfavorable gene regulatory networks present in PSC‐derived hepatocytes. In addition, the expression of essential nuclear factors such as constitutive androstane receptor, pregnane X receptor and the farnesoid X receptor were much lower in stem cell‐derived hepatocytes than in PHHs. Therefore, to further differentiate PSC‐derived hepatocytes to mature populations, the modulation of factors responsible for appropriate and inappropriate gene expression are required. Most recently, we have made progress in this space removing Matrigel™ extracellular matrix from our differentiation system and replacing this with recombinant laminins. This has resulted in improved hepatocyte maturity, organization, and phenotype 47. We believe that this offers the prospect that stem cell‐derived hepatocytes can be fabricated from GMP grade hESC lines, under defined conditions and may be close to clinical application 47.

## Conclusion

There has been tremendous progress in the development of hepatocyte differentiation systems from PSCs. Yet, hepatocyte immaturity and instability still persist. We have shown that this is not specific to stem cell‐derived hepatocytes, but also observed in adult hepatocytes cultured in vitro 48. We believe that key to solving this issue is the provision of a supportive cell niche, which ensures faithful hepatic differentiation and long‐term hepatocyte performance in vitro and in vivo.

## Author Contributions

D.S.: manuscript writing. D.C.H.: manuscript writing, financial support, final approval of manuscript.

## Disclosures of Potential Conflicts of Interest

The authors indicate no potential conflicts of interest.
